# Cysts that Still Persist: A Case Series of Cysticercosis on Histopathological Evaluation

**DOI:** 10.5146/tjpath.2020.01518

**Published:** 2021-09-15

**Authors:** Flora D Lobo, Kudurugundi Basavaraju Vatsala, Deepa Adiga A S, Sharada Rai

**Affiliations:** Department of Pathology, Kasturba Medical College, Mangalore, Manipal Academy of Higher Education, Manipal, Karnataka, India

**Keywords:** Cysticercosis, Neurocysticercosis, T. solium, Cystode, Pork

## Abstract

Cysticercus is the infective larval form of the cystode *T. solium* that causes cysticercosis. It is has been declared as one of the neglected endemic zoonoses by the WHO. Poor sanitation, and consumption of undercooked infected pork and raw vegetables contaminated by human feces are the potential sources. Cysticercosis can affect various organs. India is one of the endemic countries where the parasite is prevalent in many states. This study aimed to analyze and report cases of cysticercosis based on the histopathological diagnosis. This is a retrospective study that included cases which had been reported as cysticercosis on histopathology from 2010 to 2018 at a tertiary care hospital. The clinical presentation of these cases along with macroscopic and microscopic features were reviewed. There were six cases of cysticercosis that were diagnosed on histopathology during the study period. Among them, two cases were intramuscular lesions, three were subcutaneous, and one case was an intraventricular lesion in the brain. Three of the cases presented as cystic lesions. On histopathological evaluation, cysts were identified in four cases on macroscopy. Microscopically, the cross section of the cysticercus was seen in all six cases with associated inflammatory change. To conclude, cysticercosis can clinically present as a benign neoplastic or an inflammatory lesion. Microscopic findings dictate the diagnosis of cysticercosis, although histopathological evaluation is not common.

## INTRODUCTION

Cysticercosis is a zoonotic disease caused by the larval form, *Cysticercus cellulose* (*C. cellulose*) of the cystode *Taenia Solium (T. solium)*. It was added to the list of neglected tropical diseases by the WHO in 2010. Taeniasis is the intestinal infection caused by the adult tapeworm *T. solium*, and is the only subspecies that has major impact on humans (1). Poor personal hygiene, lack of basic sanitation, free roaming pigs, consuming contaminated food and undercooked infected pork are the routes of its spread (1,2). The ingested egg evolves into a cysticercus and can disseminate to involve various sites (3). We report six cases of cysticercosis that presented at various anatomical locations.

## CASE REPORTS

**Case 1: **A middle-aged female presented with a swelling in right arm. Clinically, it was a subcutaneous cystic lesion and was excised. Grossly, the specimen was a small translucent cyst. Microscopic examination revealed the cross section of a larva with evaginated scolex and body segment. There was dense inflammatory infiltrate composed of eosinophils, lymphocytes and histiocytes surrounding the larva.

**Case 2:** A 60-year-old woman presented with a cystic mass at the back of the neck for about six months. A cut opened, unilocular cyst measuring 1 cm in diameter was received for histopathological evaluation. Microscopically, cysticercus along with a chronic inflammatory infiltrate in the surrounding fibromuscular tissue was identified.

**Case 3**: An 8-year-old girl presented with a nodular swelling in the left arm and received a clinical diagnosis of lipoma. Macroscopically, a cyst filled with serous fluid measuring 1 cm in diameter was identified along with fibrofatty tissue bits. Sections from the cyst wall revealed the larva cysticercus. The fibroadipose tissue showed multiple palisading granuloma with a dense chronic inflammatory infiltrate.

**Figure 1:**
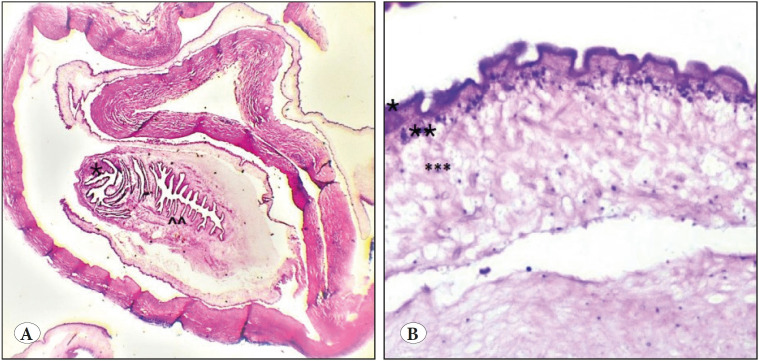
**A)** Cross section of the cysticercus, * suckers, ^^ coelomic cavity (H&E; x10). **B)** Three distinctive layers of the cysticercus: *Outer cuticular layer, ** middle cellular layer, and *** innermost reticular layer (H&E; x40).

**Case 4: **A 21-year-old lady presented with a nodule in her eyelid that was clinically suspected to be a chalazion. Excision biopsy from the nodule was sent for histopathologic examination. Macroscopically, a small, pale white nodular tissue was identified. Microscopically, a cross section of cysticercus with three different layers was seen. An outer eosinophilic corrugated cuticular layer that had microvilli with underlying bundles of muscle fibres, a middle cellular layer having small dark nuclei arranged evenly, and an innermost reticular layer consisting of loose fibrils, excretory canaliculi, and calcareous corpuscles were noted (Figure 1). A dense chronic inflammatory infiltrate was seen surrounding the parasite.

**Case 5:** A 13-year-old school going girl was found to have a right lateral ventricular lesion, when evaluated for the clinical diagnosis of central nervous system (CNS) tuberculosis. Four linear friable pale white tissue bits were received for histopathological examination. The sections revealed cysticercus, calcified bodies, areas of gliosis and eosinophilic infiltrate. It was diagnosed as neurocysticercosis.

**Figure 2:**
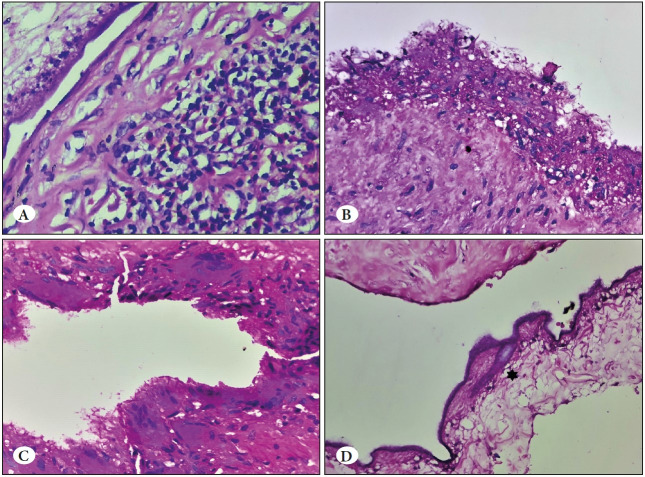
**A)** Chronic inflammation in the surrounding tissue (H&E; x40). **B)** Cyst wall lined by palisading epithelioid cells (H&E; x40). **C)** Cyst wall showing multinucleated giant cells (H&E; x40). D)* Hooklet (H&E; x40).

**Case 6:** A 33-year-old man presented to the orthopaedic department with complaint of swelling in the right arm for three months. On radiological investigation, a diagnosis of cysticercosis in the biceps muscle was rendered. Macroscopically, a cut, collapsed, white, membranous cystic structure attached to the muscular tissue was seen. On microscopy, the cysticercus larva was seen with an intramuscular extension of the cyst that was lined by a palisading histiocytic aggregate and foreign body granulomatous reaction. Dense mixed inflammatory infiltrate surrounded the cyst (Figure 2).

The clinicopathological findings are summarized in Table 1.

**Table 1:** Summary of six cases.

**Cases**	**Age/sex**	**Site and Clinical presentation**	**Microscopic findings**
1	45y/female	Subcutaneous cystic selling in the right arm	Cysticercus with dense mixed inflammation
2	60y/female	Cystic mass in the back of the neck	Cyticercus with chronic inflammation
3	8y/female	Swelling in the right arm, clinically diagnosed as lipoma	Cysticercus with multiple palisading granuloma
4	21y/female	Eyelid nodule clinically diagnosed as chalazion	Cysticercus with dense chronic inflammation
5	14y/female	Lateal ventricular lesion of the brain, clinically diagnosed as tuberculosis	Cysticercus with calcified bodies, gliosis and eosinophils
6	33y/male	Right arm swelling; radiological investigation revealed cysticercosis	Cysticercus with palisading histiocytic aggregate and foreign body granulomatous reaction

## DISCUSSION

Cysticercosis is mostly seen in those individuals who are closely associated with subsistence farming. It is common in Africa, Asia and Latin America (1). In our retrospective study, we were unable to retrieve information regarding the occupational and socioeconomic background of the patients. The case range was from an 8-year-old girl to a 60-year-old lady, and the disease affected both genders.

The infective cysticercus gets lodged at various tissues and presents as cystic mass lesions with associated clinical manifestations depending on location. Neurocysticercosis presents with the most alarming symptoms. According to the literature, the larva may persist in the CNS for long time, protected from the blood-brain barrier due to its active immune evasion mechanism, and may also elicit inflammatory changes in the surrounding tissue (4–7). In the CNS, cysts are most commonly found in the fourth ventricle, followed by the third ventricle, with the lateral ventricles being the least commonly involved (4,8). Our case of neurocysticercosis was a young girl with clinical suspicion of tuberculosis and had a lesion in the right lateral ventricle. Microscopically the host immune response was evident in the surrounding tissue.

The severity of nonneuronal cysticercosis is less on human health. Subcutaneous cysticercosis appears as painless, small, mobile nodules that are most commonly located in the arms or chest. The nodules may become swollen, tender, and inflamed which may gradually disappear. Subcutaneous cysticercosis is very common in Asia and Africa but rare in Latin America (4,9). In our study, three of the reported cases were subcutaneous lesions in the arm, back of the neck and eyelid. Based on the location, chalazion and lipoma were the provisional clinical diagnoses, respectively.

Muscular cysticercosis is frequently associated with neurocysticercosis and appears as an incidental radiological finding in the form of dot-shaped or ellipsoidal calcifications following the muscle bundles in the thighs or arms (4). Dixon reported 75% of patients with neurocysticercosis to have muscular calcifications after several years of radiographic follow-up (4,10). We have reported one case of intramuscular cysticercosis in the biceps but the CNS status of the patient was not followed up.

Macroscopically, the cysts are uniform, round or oval vesicles measuring a few millimeters to 1-2 centimeter in size. When the cysts are viable, they have a translucent membrane, through which scolices can be visualized. However, when they start degenerating, the fluid in the cyst becomes more opaque and may undergo calcification (4). In our case series, we identified intact small cysts in only two cases while in the rest of the cases it was sent after being cut opened by the operating surgeons. Microscopically all cases have shown the cross section of the parasite with variable host immune responses in the form of dense inflammation, foreign body granulomatous reaction, palisading histiocytic reactions and additionally gliosis as witnessed in the brain tissue in neurocysticercosis cases.

Among the three subspecies of* taenia, T. solium* is most commonly associated with neurocysticercosis (11,12). Histomorphologically, there are few significant differentiating features to classify these subspecies. The most easily recognizable feature is that *C. cellulosae* can have 22-32 small and large hooklets, whereas *C. bovis* (the larval form of *T. saginata*) can have none and C. viscerotropica (larval form of T. asiatica) will have only rudimentary hooks (13,14).

To conclude, in this era of advanced molecular technologies to diagnose and manage much more complicated diseases, certain preventable non-neoplastic, zoonotic diseases that are prevalent in many parts of the world deserve equal attention. With the available epidemiological data, cysticercosis is still a major health burden and cannot be underemphasized. The disease can manifest as an indolent, cystic lesion to a complicated CNS lesion. Although visualization of the parasite on histopathological evaluation is the more accurate diagnostic test, availability of less invasive and rapid diagnostic procedures saves time. India being one of the endemic countries of cysticercosis, there is ample opportunity for the investigators to learn extensively the biological nature of the parasite, the various manifestations of the disease, and its early diagnosis and management. A combined and targeted effort from the health, education and animal husbandry departments is required to educate and bring awareness to prevent the infection and its associated complications among the identified at-risk geographic population.

This is a retrospective study and only those cases that have been evaluated on histopathology were included. Most of the cysticercosis cases in clinical practice are diagnosed radiologically and might not be subjected to histopathological confirmation. Hence, the number of cysticercosis cases reported in this study may not represent the actual burden of the disease.

## Conflict of INTEREST

The authors declare no conflict of interest.

## FUNDING

None
